# Measuring mentoring in employability-oriented higher education programs: scale development and validation

**DOI:** 10.1007/s10734-023-01042-8

**Published:** 2023-05-10

**Authors:** Wendy Nuis, Mien Segers, Simon Beausaert

**Affiliations:** Department of Educational Research and Development, School of Business and Economics, Maastricht University, Tongersestraat 53, 6211 LM Maastricht, the Netherlands

**Keywords:** Employability, Competences, Higher education, Mentoring, Questionnaire

## Abstract

To keep up with technological advances and macro-economic trends, higher education has increasingly focused on developing students’ employability competences through mentoring programs. However, measuring the effectiveness of such mentoring programs has remained difficult, because many mentoring measurements are not validated or grounded in theory. Furthermore, existing questionnaires have mostly focused on one or two types of support, ignoring the wide variety of support types offered by a mentor. Therefore, the current study’s aim was to develop and validate a new questionnaire measuring various types of mentoring support. Based on a systematic literature review, a 35-item questionnaire was developed and data were collected from mentoring programs at four higher education institutions. Data were analyzed through exploratory factor analysis (*n* = 225), confirmatory factor analysis (*n* = 208), and cross-validation (*n* = 101). The results support a 6-factor model (21 items) that is statistically valid and reliable for use in universities (of applied sciences). The model includes the following factors, referring to types of support and their features: trust and availability, emotional support, networking support, autonomy support, similarity, and empathy. This questionnaire makes an original contribution insofar as (1) it is based on a sound, theoretical framework, and (2) it was demonstrated to be valid and reliable across different sub-populations in higher education. The questionnaire provides educational practitioners with a sound and valid tool to evaluate the quality of their mentoring program. It can also be used to assess what types of support could be offered to a greater extent.

## Introduction

Today, business environments and labor markets are rapidly changing due to technological advances, macro-economic trends, and the evolving knowledge-based economy (Tynjälä, 2008). This is mainly caused by the fourth industrial revolution, an era in which machine learning, artificial intelligence, and big data are prevalent elements not only in society at large, but also in higher education (Gleason, 2018). Information and knowledge transfer become more automated and the use of robotics becomes a common-day practice. Furthermore, the Covid-19 pandemic led to a shift from mere face-to-face approaches of working and teaching, to remote working and online teaching. As a result, jobs are constantly changing and new jobs are emerging at a fast pace, often partly or entirely replacing other jobs. This has an impact on the skill sets required from graduates and challenges educational institutions to deliver students with the required employability competences (Tynjälä, 2008).

Employability refers to “a set of achievements – skills, understandings and personal attributes – that makes graduates more likely to gain employment and be successful in their chosen occupations, which benefits themselves, the workforce, the community and the economy” (Yorke, 2006, p. 8). Disciplinary knowledge, transferable generic skills, emotional regulation, career development skills, self-management, and self-efficacy were the employability competences identified in a recent article integrating different conceptual views on competence-based employability in higher education (Römgens et al., 2020). Ultimately, development of the capacity to acquire and constantly update these employability competences should result in employable graduates who are well prepared to make the transition from higher education to the labor market (Donald et al., 2019), are able to cope with formal and informal work-related learning needed to master new tasks (Raemdonck et al., 2012), and are able to manage their career-building (Bridgstock, 2009).

However, these employability competences are neither innate nor easy to acquire. Hence, higher education curricula need to adopt specific instructional approaches to develop students’ employability competences. Dacre Pool and Sewell (2007) argued that in order to develop graduate employability, it is important for students to engage in a professional development trajectory in which they are provided with opportunities to reflect on learning experiences planned or undertaken. Furthermore, Jackson (2015) argued that students should engage in a continuous process of feedback and self-reflection to further develop their professional skills. For these reasons, mentoring is regarded as a suitable instructional approach, as it is precisely the mentor who stimulates students to reflect, and in turn, develop their employability competences (Lleó et al., 2018). This was confirmed by Martin et al. (2011), who researched the relationship between mentoring and employability competences. They found that mentoring nurtures employability skills development in students, as well as their ability to apply these skills. Additionally, other researchers have found positive effects of mentoring on developing soft skills (Roy & Brown, 2016), intercultural competence (Jones et al., 2019), career management skills (Bonner et al., 2019), research competency (Davis & Jones, 2017), and career decision self-efficacy (Ayoobzadeh, 2019).

For these reasons, mentoring has become an integral part of many higher educational curricula, and a wide variety of mentoring programs are currently in use (Santora et al., 2013). To measure the effectiveness of these mentoring programs, many researchers use questionnaires. However, most questionnaires show one or more of the following shortcomings (Crisp & Cruz, 2009; Gershenfeld, 2014). First, the questionnaire may not be anchored in theory. For example, many survey instruments are considered “self-developed” and lack theoretical underpinning (e.g., Lloyd & Bristol, 2006; Sorrentino, 2007). Second, the questionnaire may not be aligned with the mentoring definition applied. For example, the study by Tominaga and Kogo (2018) defined mentoring as “a mutually beneficial relationship between an e-mentor and an e-learner that provides new learning as well as career and emotional support” (p. 1777), but their survey did not include a subscale on career or emotional support. Third, the questionnaire may not be validated (Nuis et al., 2022).

Due to these prevalent shortcomings, it remains difficult to reliably measure the effects of mentoring programs. Therefore, this study aimed at developing and validating a theoretically sound instrument that takes an employability-oriented perspective. Based on systematic literature reviews on mentoring in higher education, it became clear that the supporting function of the mentor must be central (Crisp & Cruz, 2009; Nuis et al., 2022). This is in line with previous research outcomes that also highlighted the importance of mentor support (e.g., Fleck & Mullins, 2012; Holt & Fifer, 2018; Zaniewski & Reinholz, 2016). Consequently, it is exactly such a spectrum of types of support that we aim to bring together in order to create a validated questionnaire that, in turn, makes it possible to measure those different types of support and their features. Furthermore, to the best of our knowledge, this is the first instrument that brings together these different types of mentoring support based on a systematic literature review. In addition, thorough statistical analyses will be performed to evaluate the reliability and construct validity of the instrument. Hence, this validated and theoretically sound questionnaire will enable higher education practitioners to evaluate the effectiveness of their mentoring programs in order to support their graduates’ employability competences.

## Analytical framework

### Mentoring: types of support

Mentoring is known to support students in the development of their employability competences, and it has become a more prevalent educational practice within the last decade (e.g., Martin et al., 2011; Santora et al., 2013). In previous research and as a preface to this validation study, we conducted a systematic literature review to conceptualize mentoring in higher education and to shed light on the situation with regard to existing mentoring measures (Nuis et al., 2022). Based on that review, we defined mentoring as “a formalized process based on a developmental relationship between two persons in which one person is more experienced (mentor) than the other (mentee). The mentor provides support to promote and facilitate student success, competence development, and career development” (Nuis et al., 2022). Even though this literature review showed that mentoring can serve different purposes, the current study takes an employability-oriented perspective. Based on this literature review, we found that mentors’ main responsibility is providing support to their mentees (e.g., Crisp & Cruz, 2009; Nuis et al., 2022). This primary support function could be further distinguished into different types of support, such as psychosocial support, emotional support, career support, autonomy support, and networking support (e.g., Fleck & Mullins, 2012; Holt & Fifer, 2018; Zaniewski & Reinholz, 2016).

The first type of support offered by the mentor is *psychosocial support.* Psychosocial support was identified in the foundational work by Kram and Isabella (1985) as one of the most important types of support a mentor can provide. It focuses specifically on the relationship that needs to be built between the mentor and mentee. It represents the mentor’s primary task, as relationship quality and quality of interaction are considered the foundation for other types of support (e.g., Beyene et al., 2002; Eby & Robertson, 2020). In the mentoring research, psychosocial support was considered a broad and all-encompassing term; various features of psychosocial support have been identified (e.g., Beyene et al., 2002; Eby & Robertson, 2020): trust, empathy, similarity, and availability. Mentors should be perceived as *trustworthy*, especially when it comes to guiding the mentee toward the right path (Beyene et al., 2002). In addition, this feeling of trust is most often based on high levels of self-disclosure (Kram & Ragins, 2007). *Empathy* is another important feature of psychosocial support, which can be described as the cognitive capacity to understand another person’s needs, affective sensitivity to a person’s feelings, and a behavioral ability to convey understanding to a person (Shaw et al., 2012). The fundamental grounding that characterizes empathy is the feeling of respect for others, as respect lends a normative character to the dignity to which every individual is intrinsically entitled (Magrì, 2019). *Similarity* refers to a perceived similarity in attitudes, beliefs, values, or personality between the mentor and mentee, and is known to be one of the strongest predictors of mentoring relationship quality (Eby et al., 2013). Finally, mentor *availability* is an important feature stemming from attachment theory (Eby & Robertson, 2020), which prescribes that both physical and emotional availability are important to create intimacy and a high-quality relationship.

Second, *emotional support* is offered by the mentor. Emotional support is often described in conjunction with psychological support, and it focuses on the emotional feelings of the mentee (Cohen, 1995). It involves providing moral support to the mentee and identifying and discussing personal issues, difficulties, uncertainties, or fears (Crisp, 2009). Another important element of emotional support focuses on building the mentee’s self-confidence through praise and encouragement (Schockett & Haring-Hidore, 1985).

A third type is known as *career support*, an aspect of the mentoring relationship that prepares the mentee for career advancement (Noe, 1988). This aspect of the mentor–mentee relationship is found to be less intimate than psychosocial support (Fleck & Mullins, 2012). The most important activities include examining different degree options and assistance with making career-related decisions (Crisp, 2009), as well as helping mentees to network with others and offering advice (Kram, 1988). Lastly, it might involve elements of sponsorship, exposure, and visibility, in which the mentor provides mentees with opportunities to participate in projects that increase their visibility and exposes them to future career-related opportunities (Kram, 1988).

Fourth,* autonomy support* can be offered by the mentor. Autonomy support within the context of higher education can be defined as “the affirmation of the students as unique, active, and volitional individuals” (Larose et al., 2005, p. 114). This definition implies that mentors acknowledge their mentees’ perspectives and encourage them to think independently. It also means that the role of the mentor is to help mentees to make their own choices, ones that are aligned with their own norms and values (Brodeur et al., 2015). Lastly, mentors should not exert any unilateral control over choices or decisions made by the mentee.

The fifth and last type of support, *networking support* is regarded as a primary function of graduate mentoring (Fleck & Mullins, 2012). Within this type of support, mentors help their mentees to network and facilitate their access to key networks (Beyene et al., 2002). It involves helping mentees to make connections within their professional field (Tenenbaum et al., 2001). Mentors may invite and accompany their mentees to academic and/or community events, or even organize such networking activities themselves (Fleck & Mullins, 2012).

These different types of mentoring support have proven to be effective for achieving different kinds of student outcomes. For instance, a study conducted by Fleck and Mullins (2012) found significant positive relationships between psychosocial support and relational and attitudinal outcomes. Furthermore, they found significant positive relationships between career support and career-related and motivational outcomes. Lastly, Nuis et al. (2022) found a significant positive relationship between networking support and attitudinal outcomes. In addition, a study conducted by Fullick et al. (2012) found that psychosocial and career support was positively associated with stress reduction. However, in general, research on the relationship between certain types of support and outcome variables is scarce.

### Previous measures of mentoring

Our systematic literature review also demonstrated that mentoring is measured in different ways, for instance through questionnaires (*n* = *60)*, interviews (*n* = *29*), on-site measurements (*n* = *13*), or reflection journals (*n* = *11*) (Nuis et al., 2022). The findings showed that empirical studies most often used questionnaires to measure the relationship between mentoring and several outcome variables. For 52% of the questionnaires, the author(s) reported several validity and/or reliability indices, such as factor analyses and/or Cronbach’s alpha. The remaining studies did not report on the validity and/or reliability of their survey instruments used. Within the group of validated questionnaires, 20 distinct survey instruments were used and only five of them were used in more than one study. These were the College Student Mentoring Scale (Crisp, 2009; *n* = 6), Academic Mentoring Behaviour Scale (Soucy & Larose, 2004; *n* = 3), Noe’s (1988) Mentor Functions Instrument (*n* = 3), the Mentor Relationship Assessment Scale (Gullan et al., 2016; *n* = 2), and the questionnaire by Tenenbaum et al., (2001; *n* = 2).

A subsequent examination of the subscales and items revealed that most questionnaires (*n* = *5*) were used to assess the extent to which the mentees felt that support was offered to them, such as psychological and emotional support, academic subject knowledge support, psychosocial support, and career support (e.g., Allen et al., 1999; Crisp, 2009; Grant-Vallone & Ensher, 2000). However, these questionnaires mainly measured one or two types of mentor support. For instance, the Mentor Behaviour Scale by Brodeur et al. (2015) measured competency support and autonomy support, and Noe’s (1988) Mentoring Functions Instrument measured psychosocial support and career support. Consequently, this study aimed at developing and validating a questionnaire that measures a diverse but distinctive set of types of support that are all relevant when mentoring students in higher education.

## Method

### Scale construction

To construct the scale and establish content validity, the ability of the scale to appropriately measure what it intends to measure, we followed the approach by Lamm et al. (2020). They argued that one should use multiple approaches to establish content validity, such as combining a systematic literature review with an expert panel. Consequently, in the first step, we used the results of the systematic literature review by Nuis et al. (2022). Based on their review and the argumentation presented in the previous section, we assumed that the questionnaire should include multiple scales, each representing one type of support offered by the mentor. Then, we used their results to compile a list of validated questionnaires, including all subscales, items, and item loadings (Nuis et al., 2022). In a second step, we created an expert panel, consisting of three researchers with expert knowledge on mentoring in higher education, to select the items that best captured the underlying content of each scale, keeping in mind the analytical framework. For example, to create the scale for emotional support, each member of the expert panel individually extracted the five most relevant items from the item pool. To arrive at a consensus among the experts, an iterative approach was used. According to Gliddon (2006), the number of iterations should range from two to eight rounds. Within the current study, three iterations were necessary to discuss each panelist’s selected items and arrive at a consensus. During these iterations, item loadings (0.50 or higher) and theoretical relevance were both considered. In the following step, the phrasing of existing items was examined to assess whether the phrasing was suitable within the current research setting (students in higher education). If the phrasing matched well enough, they remained unchanged; otherwise, the phrasing of the items was adapted by the expert panel. In addition, new items were developed by the expert panel if (1) a scale did not consist of three or more items or (2) if the items did not yet fully cover the theoretical description. Ultimately, a consensus was reached regarding the initial questionnaire, which resulted in the following set of subscales and items.

The first subscale covered psychosocial support and consisted of 14 items in total. The item pool was consulted to extract items that covered the four features of psychosocial support: trust, empathy, similarity, and availability. The items stemmed from seven different questionnaires (Armsden & Greenberg, 1987; Cornelius et al., 2016; Crawford et al., 2014; Gullan et al., 2016; Scandura & Ragins, 1993; Tenenbaum et al., 2001; Wolfe et al., 2008). Eight items remained identical, five were adapted and one was newly developed. Where item loadings were known, all identical and adapted items had loadings above 0.5. An overview of the selected items and their respective original sources can be found in Table 1.

**Table 1:** An overview of the selected items, their original item loading, and their original source

Item		Item description	Item loading	Source
*Psychosocial support*				
PS1	Trust	I exchange confidences with my mentor	0.77	Scandura and Ragins (1993)
PS2		I trust my mentor	0.521	Armsden and Greenberg (1987)
PS3		My mentor and I achieve a high level of trust	Unknown	Cornelius et al. (2016) (adapted)
PS4	Availability	My mentor listens carefully to me	0.82	Crawford et al. (2014)
PS5		My mentor makes time for me	0.76	Gullan et al. (2016)
PS6		My mentor is easy for me to talk with	0.71	Crawford et al. (2014)
PS7		My mentor is easy to approach	Unknown	Wolfe et al. (2008) (adapted)
PS8	Similarity	My relationship with my mentor is personal	0.81	Gullan et al. (2016)
PS9		I speak to my mentor like I would to a friend	0.81	Gullan et al. (2016)
PS10		My mentor is someone I could be friends with	0.75	Gullan et al. (2016) (adapted)
PS11		My mentor displays values similar to my own	0.75	Tenenbaum et al. (2001) (adapted)
PS12	Empathy	My mentor conveys feelings of respect for me	0.81	Tenenbaum et al. (2001) (adapted)
PS13		My mentor respects my feelings	0.714	Armsden and Greenberg (1987)
PS14		My mentor and I have a respectful relationship		NEW
*Emotional support*				
ES1		My mentor congratulates me when I do something right	0.930	Brodeur et al. (2015)
ES2		My mentor provides emotional support	0.81	Crawford et al. (2014)
ES3		My mentor advises me in relation to personal problems	0.79	Crawford et al. (2014)
ES4		My mentor often tells me what I do well	0.787	Brodeur et al. (2015)
ES5		My mentor encourages me to talk openly about my anxiety and fears	0.75	Tenenbaum et al. (2001) (adapted)
ES6		My mentor helps me to talk about my difficulties	0.74	Armsden and Greenberg (1987)
ES7		I share personal problems with my mentor	0.73	Scandura and Ragins (1993)
ES8		I tell my mentor about my troubles	0.726	Armsden and Greenberg (1987) (adapted)
*Career support*				
CS1		My mentor devotes special time and consideration to my future career	0.73	Scandura and Ragins (1993) (adapted)
CS2		My mentor takes a personal interest in my future career	0.71	Scandura and Ragins (1993) (adapted)
CS3		My mentor explores future career options with me	0.67	Tenenbaum et al. (2001) (adapted)
CS4		My mentor helps me make connections between my studies and professional practice	0.66	Tenenbaum et al. (2001)
*Autonomy support*				
AS1		I feel that my mentor provides me choices and options	0.79	Williams and Deci (1996)
AS2		My mentor encourages me to ask questions	0.69	Williams and Deci (1996)
AS3		My mentor listens to how I would like to do things	0.70	Williams and Deci (1996)
AS4		My mentor tries to understand how I see things before suggesting a new way to do things	0.71	Williams and Deci (1996)
*Networking support*				
NS1		My mentor offers me to introduce me to others who can provide me with opportunities	Unknown	Fullick et al. (2012)
NS2		My mentor connects me to colleagues, both in school and the professional field	Unknown	Fullick et al. (2012) (adapted)
NS3		My mentor encourages me to identify the strengths and weaknesses of my network		NEW
NS4		My mentor supports me in developing my own network		NEW
NS5		My mentor offers the possibility to make use of his/her network		NEW

The second subscale focused on emotional support and consisted of eight items. The item pool was consulted to extract items that covered the theoretical description of emotional support. The items stemmed from the questionnaires by Brodeur et al. (2015), Crawford et al. (2014), Scandura and Ragins (1993), Armsden and Greenberg (1987), and Tenenbaum et al. (2001). Six items stayed identical, and two items were adapted; all of these items had loadings above 0.5.

The third subscale concerned career support and consisted of four items. Four theoretically relevant items were extracted from the item pool. Two items were from the questionnaire by Scandura and Ragins (1993) and two items were from the questionnaire by Tenenbaum et al. (2001). Three items were adapted by the research team to better match the research context, and one item remained identical to the original source.

The fourth subscale on autonomy support was not based on any of the items found in the systematic literature review (Nuis et al., 2022). The items from the item pool that assessed autonomy support did not cover the theoretical description of the concept or did not have item loadings above 0.50. Therefore, the autonomy support scale was based on an existing scale by Williams and Deci (1996). The four items that best suited the theoretical description of autonomy support as part of mentoring and had the highest loadings were selected to be part of the scale. No adaptations to the phrasing were needed.

The fifth scale focused on networking support and consisted of five items in total. Two items were extracted from the item pool and came from the questionnaire by Fullick et al. (2012). However, a minimum of three items per scale is recommended to yield reliable solutions during a factor analysis (Marsh et al., 1998). Therefore, three new items were developed by the research team that matched the theoretical definition of networking support.

### The questionnaire

The process of scale construction eventually led to the development of the Mentoring Support Scale. This questionnaire contained 35 items, divided over 5 subscales: 20 original items and 15 new items (11 based on previous items and four completely new items). For the final questionnaire, all scales were introduced by the following sentence: “To what extent do you agree with the following statements?” Additionally, the statements were evaluated using a 5-point Likert scale ranging from *strongly disagree* (1) to *strongly agree* (5). Lastly, the questionnaire included four items asking about demographic information, such as gender, age, nationality, and program of study.

### Procedure and participants

Data were collected using the newly developed measure (titled the Mentoring Support Scale) from undergraduate students at two universities of applied sciences in the Netherlands and one university of applied sciences in Belgium. Additional data for cross-validation were collected from graduate students at one university in the Netherlands, to make sure the questionnaire would fit a university context as well as a university of applied sciences context. All educational institutions offered academic mentoring programs to their students. These mentoring programs consisted of several similar features: (1) mentoring provided individually during one academic year, (2) a focus on the development of students’ (employability) competences, such as working in teams, written and verbal communication, and self-efficacy, and (3) the exclusion of guidance on academic matters (e.g., remedial teaching activities). Because of this context, the Mentoring Support Scale is validated taking an employability perspective, even though its initial development took a broader view on potential mentoring outcomes. Most students in these mentoring programs were either in teacher training or studied hotel management.

Program leaders of these mentoring programs were contacted and a meeting between the executive researcher and each program leader took place. During these meetings, the program leaders were informed of the subject of the study and were invited to participate. All program leaders decided to participate, and therefore they became the first point of contact. All students who participated in the mentoring programs were informed about the research study by their program leaders and were asked to participate in the study voluntarily. They were invited to fill out an online questionnaire along with an informed consent form. The questionnaire was presented in English and data collection took place in June 2020 and June 2021, as one round of data collection did not yield enough data to conduct the necessary analyses. In total, 281 students completed the survey in June 2020 and 253 students in June 2021. Most of the sample (79.8%) was female, which is in line with the percentage of female employees working in the respective disciplines (primary school teachers at 80% female and hospitality employees at 57% female; CBS, 2020). The average age was 23.5 years. A more detailed overview of the collected data is presented in Table 2.

**Table 2:** Detailed overview of collected data

	Educational institution	Number of participants	Used for:
June 2020	Dutch university of applied sciences (1)	120	EFA
	Dutch university of applied sciences (2)	24	
	Belgian university of applied sciences	81	
	Dutch university	56	Cross-validation
	**Sub-total**	**281**	
June 2021	Dutch university of applied sciences (1)	78	CFA
	Dutch university of applied sciences (2)	74	
	Belgian university of applied sciences	56	
	Dutch university	45	Cross-validation
	**Sub-total**	**253**	
	**Total**	**534**	

### Data analysis

#### Exploratory factor analysis

The data collected at three universities of applied sciences (*n* = 225) in June 2020 were used to conduct an exploratory factor analysis (EFA) in SPSS 26.0. An EFA is “an exploratory method used to generate theory; researchers use EFA to search for the smaller set of k latent factors to represent the larger set of j variables” (Henson & Roberts, 2006, p. 395). To perform the EFA adequately, three steps were taken.

First, the suitability of the sample was assessed by looking at the number of respondents per item, the correlation matrix, Bartlett’s test of sphericity (Bartlett, 1954), and the Kaiser–Meyer–Olkin (KMO) measure of sampling adequacy (Kaiser, 1970).

Second, the factors were extracted from the data. As the aim of this study was not to reduce the data but to construct factor solutions that are clearly interpretable while still providing a good fit to the data, maximum likelihood estimation was selected as the most appropriate factor extraction method (Heeler et al., 1977). In addition, the maximum likelihood estimation is considered robust, so it controls for potential non-normality of the data (Fuller & Hemmerle, 1966). Furthermore, previous research recommended that multiple criteria should be used for factor extraction (e.g., Henson & Roberts, 2006). Therefore, this study also used the results of the scree plot and Kaiser’s recommendation of eigenvalues over 1 to determine the number of extracted factors (Field, 2000).

Third, an oblimin rotation was chosen because it was expected that the subscales of the latent constructs were intercorrelated, and this rotation method allowed for such correlations (Pallant, 2010). Furthermore, the internal consistency and reliability of the subscales were calculated using Cronbach’s alpha coefficients (DeVellis, 2003).

#### Confirmatory factor analysis

While EFA is used to generate theory, confirmatory factor analysis (CFA) is based on pre-existing theory (Schreiber et al., 2006). Based on the factor structure that resulted from the EFA, Amos 24.0 was used to conduct a CFA on a second sample to determine the theory’s validity. By doing so, the structural relationships between the different items and their latent variables were analyzed, as well as the intercorrelations between the variables. The data for this analysis came from the same three schools and were collected in June 2021 (*n* = 208).

To examine the extent to which the proposed model fit the data, four goodness-of-fit measures were used. First, the chi-square/df ratio (χ^2^/df) was calculated to provide information on model parsimony. According to Tabachnick et al. (2007), parsimonious models have a χ^2^/df value between 1 and 3. Second, the comparative fit index (CFI) was examined to compare the specified model with the base model. The cut-off value for the CFI is > 0.9 (Casanova et al., 2019). Third, the root mean square error of approximation (RMSEA) was determined to evaluate the overall model fit while taking into account the complexity of the model. RMSEA values < 0.08 indicate acceptable model fit (Wang & Wang, 2012). Fourth, the standardized root mean square residuals (SRMR) index was calculated to analyze the average of standardized residuals between the observed and the hypothesized covariance matrices. An SRMR value between 0 and 0.08 is regarded as an indicator of good model fit (Hu & Bentler, 1999).

#### Cross-Validation

Cross-validation was performed using data from a Dutch university (*n* = 101). Three tests of measurement invariance were conducted to assess the psychometric equivalence of the latent construct across different groups (Putnick & Bornstein, 2016). *Configural invariance* tests whether the construct has the same pattern of factors and loadings across different groups, that is, equivalence of model form. *Metric invariance* tests whether each item contributes to the latent construct to a similar degree across groups, that is, equivalence of item loadings on factors. *Scalar invariance* tests whether mean differences in the latent construct capture all mean differences in the shared variance of the items, that is, equivalence of item intercepts.

## Results

### Exploratory factory analysis

A first step was to determine if the sample (*n* = 225) was large enough. A traditional rule of thumb is to have at least 5–10 respondents per item (Nunnally, 1978). Other researchers have argued that a sample size of 225 is fully adequate to conduct an EFA (Sapnas & Zeller, 2002). Furthermore, the correlation matrix showed many coefficients larger than 0.3, which provided evidence of strong enough intercorrelations among items to proceed with a factor analysis (Tabachnick et al., 2007). The VIF values were all below 5, indicating that there was no severe risk of multicollinearity (Daoud, 2017). Bartlett’s sphericity test (1954) yielded a χ^2^ value of 4841.319 and reached statistical significance at the *p* < 0.001 level. The KMO value was 0.923, thereby exceeding the recommended value of 0.6 (Tabachnick et al., 2007). Since all measures supported the suitability of the sample for a factor analysis, an exploratory factor analysis of the 35 items of the Mentoring Support Scale was performed.

**Fig. 1:**
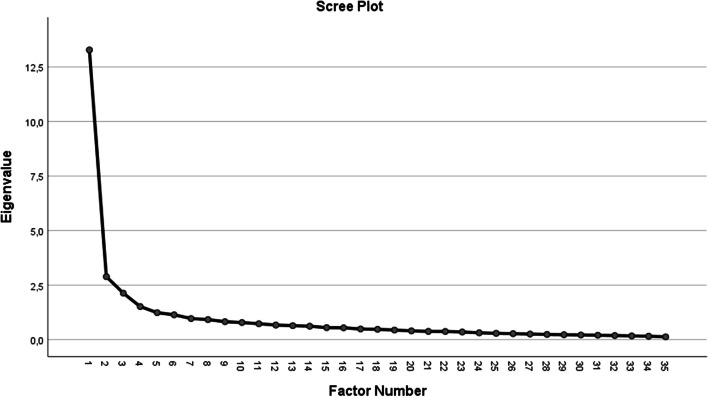
Scree plot for all 35 items from the original scale

The factor analysis revealed six components with eigenvalues exceeding 1, explaining 37.9%, 8.2%, 6.1%, 4.3%, 3.5%, and 3.3% of the variance, respectively, giving 63.4% total explained variance. The scree plot for this factor solution is presented in Fig. 1, which shows that the last inflection point occurred at the seventh factor. Therefore, it was concluded that the factor structure consisted of six distinct factors.

The pattern matrix, presented in Table 3, shows the distribution of items and their factor loadings across the six identified factors. Item loadings below 0.4 were suppressed and cross-loadings were excluded. The results of the EFA left a measure consisting of six scales made up of 23 items.

**Table 3:** Pattern matrix for the Exploratory Factor Analysis of the Mentoring Support Scale

Item	Factor loading					
	1	2	3	4	5	6
*Factor 1—trust and availability (α* = *0.890)*						
PS1	0.775					
PS2	0.732					
PS3	0.696					
PS5	0.509					
PS6	0.462					
*Factor 2—emotional support (α* = *0.830)*						
ES7		-0.957				
ES8		-0.935				
ES3		-0.463				
*Factor 3—networking support (α* = *0.833)*						
NS1			0.843			
NS2			0.734			
NS5			0.636			
NS4			0.543			
NS3			0.486			
*Factor 4—autonomy support (α* = *0.812)*						
AS3				0.654		
AS2				0.603		
AS1				0.495		
AS4				0.441		
*Factor 5—similarity (α* = *0.774)*						
PS10					0.881	
PS9					0.610	
PS11					0.480	
*Factor 6—empathy (α* = *0.861)*						
PS13						-0.723
PS12						-0.579
PS14						-0.508

*Note. n* = *225. The extraction method was maximum likelihood with oblimin rotation (with Kaiser normalization). Factor loadings with absolute values below 0.4 were suppressed*

First, the psychosocial support variable originally consisted of four features that captured the full range of psychosocial support. However, the exploratory factor analysis showed that this variable resolved into three separate factors: trust and availability, similarity, and empathy. The first scale that emerged from the EFA contained items that originally belonged to the features of trust and availability. Therefore, this first factor was renamed trust and availability, and consists of 5 items. Example items are “My mentor and I achieve a high level of trust” and “My mentor is easy for me to talk with.” Items PS4 and PS7 had item loadings lower than 0.4 and were therefore suppressed.

The second factor was initially hypothesized as emotional support. The factor analysis resulted in three items remaining in the scale, as the other five items had factor loadings below 0.4. These three items are “My mentor advises me in relation to personal problems”, “I share personal problems with my mentor” and “I tell my mentor about my troubles.” Since all three items revolve around sharing personal issues with the mentor, the name of this second factor was retained as emotional support.

The third factor was initially hypothesized as networking support. The factor analysis showed that all five networking support items should be retained and fell into the same scale. Therefore, this scale kept its original name, networking support. Example items are “My mentor encourages me to identify the strengths and weaknesses of my network” and “My mentor supports me in developing my own network.”

The fourth factor was initially hypothesized as autonomy support. The factor analysis showed that all four items for this variable fell into the same scale and had factor loadings above 0.4. Therefore, this factor was named autonomy support. Example items are “I feel that my mentor provides me choices and options” and “My mentor tries to understand how I see things before suggesting a new way to do things.”

The fifth factor included items that originally belonged to the similarity feature of psychosocial support. As the results of the factor analysis demonstrated that the students viewed this feature as a separate mentoring function, similarity was regarded as the fifth factor of the questionnaire. This factor contains three items with item loadings above 0.4. Example items are “I speak to my mentor like I would to a friend” and “My mentor displays values similar to my own.”

The sixth factor also emerged as a new factor from the exploratory factor analysis. Similar to the previous results, empathy was originally one of the features of the psychosocial support variable. However, the analysis revealed that students viewed this construct as a separate mentoring function, instead of belonging to psychosocial support. Therefore, empathy was identified as the sixth factor, containing three items with item loadings above 0.4. Example items are “My mentor conveys feelings of respect for me” and “My mentor and I have a respectful relationship.” All scales had Cronbach’s alphas higher than 0.70 (see Table 3).

Lastly, career support did not end up as a separate factor during the exploratory factor analysis. The career support items had loadings below 0.4, which is the reason why they were suppressed during the analysis. Furthermore, the loadings for factor 2 and factor 6 are shown to be negative. According to Field (2000), this is due to factor score indeterminacy, which is especially the case in exploratory factor analyses. The sign of the factor loading is indeterminate and factor loadings in EFA are considered absolute values.

### Confirmatory factor analysis

**Fig. 2:**
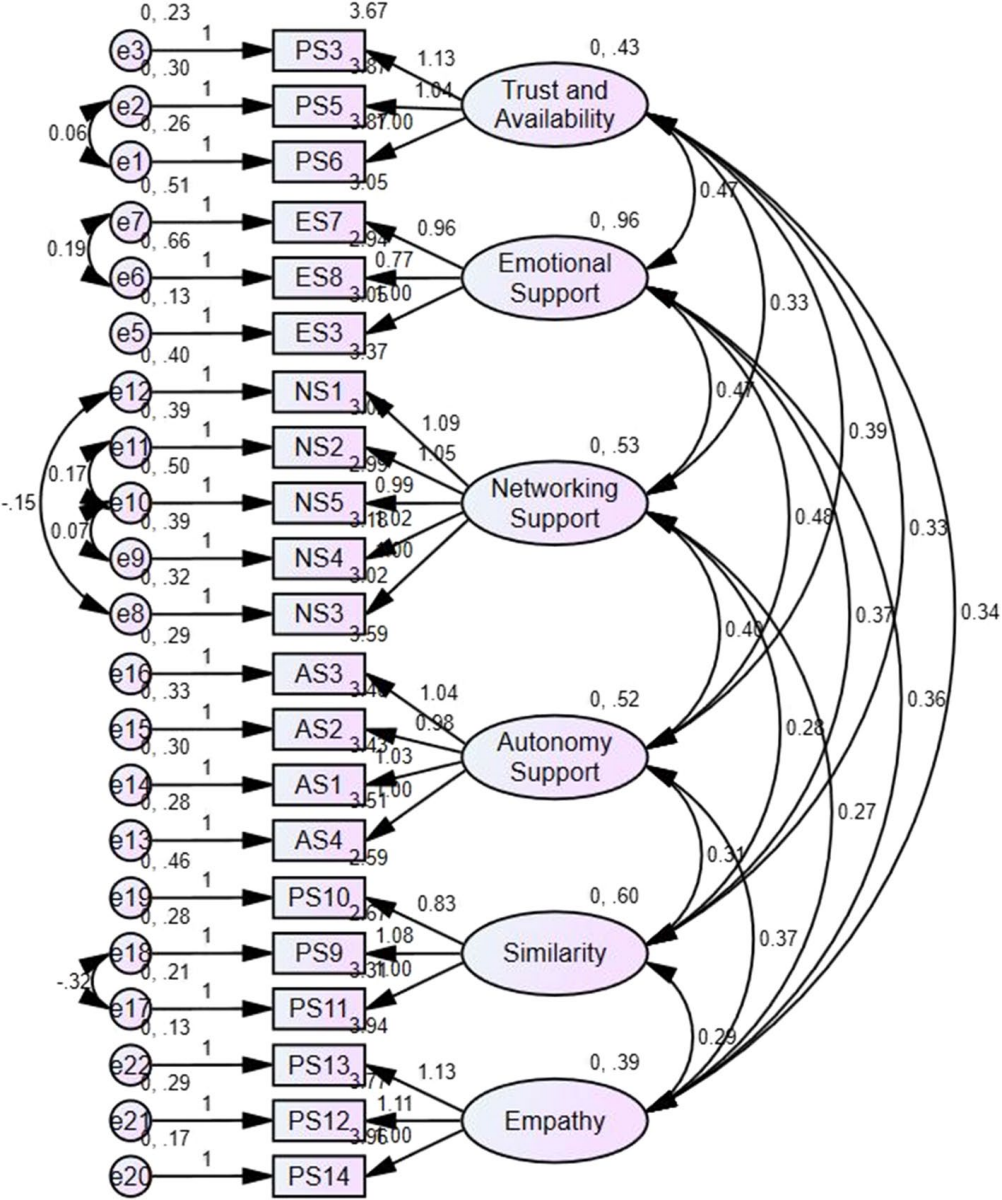
Confirmatory factor analysis

A confirmatory factor analysis was performed to confirm the questionnaire’s construct validity. The CFA was conducted on the data collected in June 2021 at the same universities of applied sciences (*n* = 208). The results are presented in Fig. 2. Based on the modification indices, items PS1 and PS2 were excluded and six relations between error terms were added to increase the model fit. The results of the four goodness-of-fit measures are as follows. First, the chi-square/df ratio was between 1 and 3 (χ^2^/df = 2.302). Second, the CFI was above 0.9 (0.926). Third, the RMSEA was below 0.08 (0.079, CI: 0.069–0.090) and the SRMR was between 0 and 0.08 (0.0514). Therefore, all fit indices demonstrated a good model fit. The reliability of the scales was calculated on this subsample as well, and all Cronbach’s alphas were higher than 0.70 (Table 4).

**Table 4:** Descriptive statistics and reliability indices for confirmatory factor analysis

Scale	*Items*	*M*	*SD*	*α*
Trust and availability	3	3.80	0.86	0.859
Emotional support	3	3.01	1.12	0.868
Networking support	5	3.12	0.98	0.879
Autonomy support	4	3.48	0.92	0.877
Similarity	3	2.86	0.94	0.758
Empathy	3	3.89	0.81	0.878

*Note. n* = *208*

### Cross-validation

**Fig. 3:**
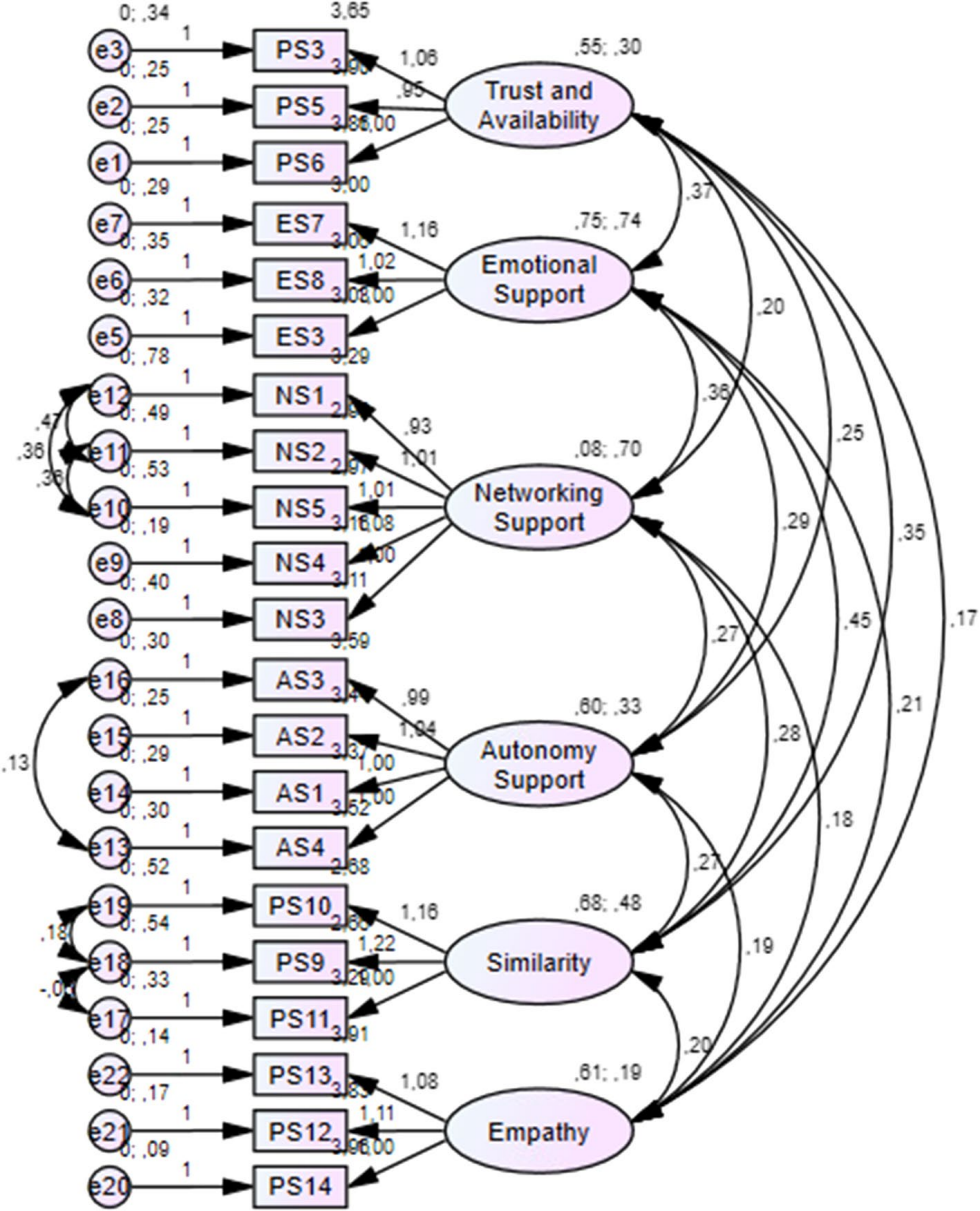
Cross-validation

To assess the psychometric equivalence of the questionnaire in a university context, cross-validation was conducted on a third dataset (*n* = 101). The results are presented in Fig. 3. To increase model fit, some adaptations were made regarding the relations between error terms. Four relations were deleted (e1-e2, e6-e7, e8-e12, e9-e10) and four relations were added (e11-e12, e10-e12, e13-e16, e18-e19). The goodness-of-fit indices showed a good model fit. First, the chi-square/df ratio was between 1 and 3 (χ^2^/df = 1.680). Second, the CFI was above 0.9 (0.923). Third, the RMSEA was 0.08 (CI: 0.065–0.099) and the SRMR was between 0 and 0.08 (0.0623). The reliability of the scales was calculated on this subsample as well, and all Cronbach’s alphas were higher than 0.70 (Table 5).

**Table 5:** Descriptive statistics and reliability indices for cross-validation

Scale	*Items*	*M*	SD	α
Trust and availability	3	4.36	0.77	0.765
Emotional support	3	3.84	1.07	0.885
Networking support	5	3.09	1.07	0.924
Autonomy support	4	4.09	0.78	0.839
Similarity	3	3.67	1.03	0.827
Empathy	3	4.55	0.58	0.832

*Note. n* = *101*

Additionally, three tests of measurement invariance were conducted. The configural invariance test showed a CFI of 0.914, thereby indicating that the model has configural invariance (equivalence of model form across groups). Furthermore, the difference in CFI between model 1 (0.914) and model 2 (0.912) was 0.002 (ΔCFI ≤ 0.01) and the difference in Mc NCI between model 1 (0.541) and model 2 (0.522) was 0.009 (ΔMc NCI ≤ 0.02). Therefore, the results indicate that the model reached metric invariance, that is, equivalence of item loadings on factors (Cheung & Rensvold, 2002). The third test concerns scalar invariance, assessing the equivalence of item intercepts. The results showed a ΔCFI of 0.022 and a ΔMc NCI of 0.078, which means that the model did not reach scalar invariance.

## Discussion

### Discussion of findings

Technological advances and macro-economic trends have resulted in a fast-changing labor market in which possessing employability competences, such as working in teams, verbal and written communication, and self-efficacy, is of utmost importance. This is true not only for employees working in today’s society, but also for students in higher education preparing themselves to make the transition from higher education to the labor market. To successfully make that transition, students should become lifelong learners who are capable of reflecting on their competence development, as reflective abilities are known to be key to employability. Therefore, mentoring is often used as a pedagogical approach, as it is precisely the mentor and the mentoring tools that stimulate students to reflect, and in turn develop their employability competences. However, measuring the effectiveness of such mentoring programs has remained difficult and flawed, because many mentoring measurements have shown methodological shortcomings such as the lack of a conceptual underpinning and/or an absence of or weak validity scores (Crisp & Cruz, 2009; Gershenfeld, 2014; Nuis et al., 2022). Furthermore, existing questionnaires have mostly taken a limited view of the types of support measured. Therefore, this study reports the development of a questionnaire that is anchored in theory and aimed at including a more all-encompassing view of the types of support necessary for successful mentoring, which was validated using advanced statistical analyses. As a result, our questionnaire is one of the first that is based on a sound, theoretical framework, and has been demonstrated to be valid and reliable across different subpopulations of students. In this way, the questionnaire can be used to measure the effectiveness of mentoring programs in higher education, especially the ones that take an employability oriented-perspective.

This study followed up on a previously conducted systematic literature review (Nuis et al., 2022) by developing a measure and conducting factor analyses and a cross-validation. This approach resulted in a questionnaire containing 21 items, spread across six different scales. These scales are psychosocial support-trust and availability (3 items), emotional support (3 items), networking support (5 items), autonomy support (4 items), psychosocial support-similarity (3 items), and psychosocial support-empathy (3 items). All scales were found to be valid and reliable. The confirmatory factor analysis confirmed the questionnaire’s six-factor structure. The analysis demonstrated a good model fit, showing that the data fit the hypothesized measurement model that resulted from the exploratory factor analysis. The subsequent cross-validation, in which the measurement model was tested in an academic context as opposed to Bachelor’s programs in applied sciences, showed the robustness of the questionnaire, since the results again demonstrated a good model fit.

First, and in line with expectations, mentees made a distinction between the different types of support identified in previous research by Fleck and Mullins (2012), Holt and Fifer (2018), and Zaniewski and Reinholz (2016), among others. Moreover, the scales showed high internal reliability scores. Emotional support focuses on the role of the mentor in helping the mentee deal with personal issues or problems. Networking support refers to the guidance of a mentor in identifying and developing the mentee’s network and providing access to the mentor’s own network or potential other networks that could be of interest to the mentee. Autonomy support refers to the mentor’s acknowledgment that the mentees are independent individuals who make their own choices and decisions, without feeling that the mentor is exerting control over them. This type of support enables students to take ownership over which employability competences to develop and how to develop them.

Second, and also in line with theory, mentees do make a distinction between the key features of psychosocial support (trust and availability, similarity, and empathy). As a result, the core features of psychosocial support were identified as separate factors. More specifically, the psychosocial support construct resolved into three subscales. Trust and availability refers to a mentoring relationship in which the mentor and mentee achieve a high level of mutual trust, and in which the mentor is easy to talk to and makes time for the mentee. Similarity refers to the idea that the mentor and mentee display similar values and that the mentee can identify with the mentor as if they were friends. Empathy refers to the mutual respect the mentor and mentee show towards each other, and the fact that the mentor respects the feelings of the mentee.

Third, and in contrast to what was expected, career support was not recognized as a separate type of support by the students. Career support refers to mentoring behaviors that prepare mentees for career advancement (Noe, 1988), help them with examining different degree options, and assist them with making career-related decisions (Crisp, 2009). However, this finding could imply that students do not perceive career support to be a substantial mentoring function. This might be related to how much career support is actually offered in the investigated mentoring programs. Is it the case that career support is hardly offered and thus not recognized by the participants? Or does career support look different in mentoring programs in higher education, as opposed to career support in mentoring at the workplace? Nevertheless, the literature argued that career support is important for graduates’ school-to-work transition (Renn et al., 2014). Therefore, it would be highly interesting for future research to further explore what kind of career support is offered in such mentoring programs and how this could be measured in a relevant manner. One potential avenue would be to first explore qualitatively what the role and representation of career support is within mentoring programs in higher education, for example, through interviews and observations.

### Limitations and suggestions for future research

This study’s limitations and avenues for future research are as follows. First, the Mentoring Support Scale has been validated in one particular context, being a Western-European country adopting a mentoring program aimed at increasing students’ employability competences. Therefore, future research is needed to perform various cross-validations to be able to better generalize the results to other cultures or mentoring outcomes. Second, adaptations needed to be made regarding the relations between error terms when performing the cross-validation. This indicates that the measurement model was not fully identical across the two different groups of students, namely, applied sciences versus a university context. Future research might look into potential reasons why these error terms needed to be adapted to better suit the academic context. Second, when performing the cross-validation, the model did not reach scalar invariance. It is important to test for scalar invariance to assess if the mean differences in the latent construct capture all mean differences in the shared variance of the items (Putnick & Bornstein, 2016). However, the purpose of the current validation study was to create and validate an instrument that would behave the same across different groups, as opposed to comparing different groups. A recommendation for future research would be to investigate the source of non-invariance by sequentially adding or releasing item intercept constraints and subsequently retesting the measurement model until partial scalar invariance is achieved (Putnick & Bornstein, 2016). Lastly, future research might use the questionnaire and its different types of support to study both antecedents and outcomes of mentoring. Potential antecedents could be either environment-related or person-related, such as the influence of mentor training, learning climate, and supervision (environment), as well as personality, experience, and motivation (person). Potential outcomes of mentoring that could be studied in depth are students’ reflective abilities, employability competences, mobility, academic performance, or career orientation. When studying the relation with employability, researchers might have to take into account that employability might be differently conceptualized by schools and students.

### Practical implications

The Mentoring Support Scale can be used by higher education institutions, for example, by coordinators of mentoring programs, to effectively and reliably measure the quality of their mentoring program. This is especially the case for mentoring programs targeted at increasing student’s employability competences, because the questionnaire was validated in such a context. When employing a longitudinal research design, program coordinators could use the questionnaire to reveal the impact of the mentoring program on their student’s employability competences, or on other student learning outcomes after performing a careful cross-validation. In addition, the measurement instrument offers a nuanced understanding of the different support types that are important when mentoring for student employability. This is important for program coordinator and mentors alike, as it provides more detailed information about which support types are perceived to be more or less present within the mentor and mentoring program. From this kind of information, mentors and program coordinators can make informed decisions on how to improve their mentoring program or how to train the respective mentors.

## Conclusion

To conclude, our development and validation efforts resulted in the Mentoring Support Scale; a questionnaire containing 21 items, spread across six different scales. All scales were found to be valid and reliable, and the subsequent cross-validation showed the robustness of the questionnaire across different higher education contexts. Furthermore, and in line with expectations, mentees made a distinction between the different types of support. For psychosocial support, they made a distinction between the three key features (trust and availability, similarity, and empathy). Lastly, and in contrast to what was expected, career support was not recognized as a separate type of support by the students. However, this finding could imply that students do not perceive career support to be a substantial mentoring function of mentoring programs in higher education.

## Data Availability

Dataset available upon request to Wendy Nuis.
